# Comparative genomic analysis of mouse and human mammary tumors

**DOI:** 10.1186/gb-2011-12-s1-i12

**Published:** 2011-09-19

**Authors:** Charles M Perou

**Affiliations:** 1 Lineberger Comprehensive Cancer Center, 450 West Drive, CB# 7295, The University of North Carolina at Chapel Hill, Chapel Hill, NC 27599, USA

## 

Human breast cancer is a heterogeneous disease consisting of at least five molecular subtypes. With our increasing understanding of the genetic underpinnings of human breast cancer subtypes, many genetically engineered mouse models (GEMMs) have been created to mimic these subtypes. Traditionally, these GEMMs have been analyzed individually; however, when consolidated into a single dataset, these models have an increased sensitivity for detecting significant overlap with human subtypes. These associations between GEMMs and human subtypes can provide insight into the genetic alterations associated with the human subtypes and can provide a translational resource for preclinical drug testing. We previously performed an analysis of 13 GEMMs [1] and have since expanded our dataset to include 29 murine models. Using DNA expression microarrays and unsupervised hierarchical clustering, we identified 17 distinct murine classes from this set of 29 models. After comparison with the human subtypes by using gene set analysis, we found that seven of our classes show statistically significant overlap with five human subtypes. Although we observed no statistical overlap with the human luminal B subtype, the MMTV-NeuPyMT class shows significant overlap with the combined luminal A and B subtypes. Some of the new models fall into classes that have been defined previously, but many, such as the AIB1 and ETV6 murine models, are associated with new groups. The AIB1 and ETV6 models fall into their own classes, and each shows statistically significant overlap with HER2-enriched tumors, a subtype that was not previously observed to overlap with any GEMM. These expanded analyses have identified new and important models and are laying the groundwork for additional studies focused on DNA copy number changes and mutation status similarities between mice and humans.
